# Noninvasive Stimulation of the Ventromedial Prefrontal Cortex Indicates Valence Ambiguity in Sad Compared to Happy and Fearful Face Processing

**DOI:** 10.3389/fnbeh.2019.00083

**Published:** 2019-05-16

**Authors:** Constantin Winker, Maimu A. Rehbein, Dean Sabatinelli, Mira Dohn, Julius Maitzen, Kati Roesmann, Carsten H. Wolters, Volker Arolt, Markus Junghoefer

**Affiliations:** ^1^Institute for Biomagnetism and Biosignalanalysis, University of Muenster, Muenster, Germany; ^2^Otto Creutzfeldt Center for Cognitive and Behavioral Neuroscience, University of Muenster, Muenster, Germany; ^3^Department of Psychology and BioImaging Research Center, University of Georgia, Athens, GA, United States; ^4^Department of Psychiatry, University of Muenster, Muenster, Germany

**Keywords:** emotion, brain stimulation, tDCS, MEG, EEG, faces

## Abstract

The ventromedial prefrontal cortex (vmPFC) is known to be specifically involved in the processing of stimuli with pleasant, rewarding meaning to the observer. By the use of non-invasive transcranial direct current stimulation (tDCS), it was previously possible to show evidence for this valence specificity and to modulate the impact of the vmPFC on emotional network processing. Prior results showed increased neural activation during pleasant relative to unpleasant stimulus processing after excitatory compared to inhibitory vmPFC-tDCS. As dysfunctional vmPFC activation patterns are associated with major depressive disorder (MDD), tDCS of this region could render an attractive application in future therapy. Here, we investigated vmPFC-tDCS effects on sad compared to happy face processing, as sad faces are often used in the study of mood disorders. After counterbalanced inhibitory or excitatory tDCS, respectively, healthy participants viewed happy and sad faces during magnetoencephalography (MEG) recording. In addition, tDCS effects on an interpretational bias of ambiguous happy-sad face morphs and an attentional bias of a dot-probe task with happy and sad faces as emotional primes were investigated. Finally, in conjoint analyses with data from a previous sibling study (happy and fearful faces) we examined whether excitatory vmPFC-tDCS would reveal a general increase in processing of pleasant stimuli independent of the type of unpleasant stimuli applied (sad vs. fearful faces). MEG and behavioral results showed that happy faces promoted a relative positivity bias after excitatory compared to inhibitory tDCS, visible in left orbitofrontal cortex and in the emotion-primed dot-probe task. A converse pattern in the MEG data during sad face processing suggests the possible involvement of an empathy network and thus significantly differed from neuronal processing of fearful face processing. Implications for the bearing of vmPFC modulation on emotional face processing and the impact of specific unpleasant face expressions are discussed.

## Introduction

Accurate processing of facial expressions is essential to the evaluation of the motivations, feelings, and intentions of others. Emotional face processing is realized *via* specific cortical connections within the first few 100 ms of exposure. Involved in this network of face processing are extrastriate cortex, inferior temporal fusiform gyrus and superior temporal sulcus (STS), which show enhanced activation for emotional expressions (for review, see Haxby et al., 2000; Britton et al., 2006; Sabatinelli et al., 2011) coupled with the amygdala (for review, see Vuilleumier and Pourtois, 2007). Another region that plays a crucial role in emotion, as well as reward processing, is the ventromedial prefrontal cortex (vmPFC). The vmPFC is a major dopaminergic hub in the processing of pleasant and rewarding stimuli (for review, see Myers-Schulz and Koenigs, 2012). This pleasant valence specificity seems to be independent of the presented stimulus type as it has been shown for, e.g., emotional pleasant scenes (Sabatinelli et al., 2007), imagery of pleasant events (Costa et al., 2010), pleasant words (Keuper et al., 2013), safety signals in context of aversive conditioning (Milad et al., 2007), and monetary reward (Knutson et al., 2003; Carlson et al., 2011). An imbalance of activity in these anterior areas can cause dysfunctional behavioral symptoms and is known as crucial factor in various emotional psychiatric disorders such as social phobia (Laeger et al., 2014), posttraumatic stress disorder (Milad et al., 2009), general anxiety disorder (Greenberg et al., 2013), dysphoria (Sabatinelli et al., 2015), or major depressive disorder (MDD; Pizzagalli et al., 2001). Thus, the recovery of such imbalance could help decreasing symptom severity. In many such cases toward this goal, pharmacotherapy is a recommended treatment. However, due to frequent accompanying side effects in psychopharmacological interventions, non-invasive stimulation technologies might offer an appealing substitute or even alternative in the future. For example, transcranial direct current stimulation (tDCS) has attracted vast attention in the last two decades, as it is inexpensive, easily applicable, and due to its mobility usable “everywhere, everytime.” tDCS operates *via* at least two electrodes (one anode, one cathode) that are applied to the skin in order to establish a closed circuit of direct current. The current flow from one electrode to the other affects the traversing brain tissue (for a detailed description, see Paulus, 2014). Furthermore, the current below the anode has been reported to increase excitability of neurons, whereas the reversed current under the cathode results in decreased excitability (Nitsche et al., 2008).

In the framework of a valence specificity of vmPFC, application of tDCS might be able to systematically increase or decrease processing of pleasant content. In fact, in three previous studies, we found evidence for a successful modulation of such pleasant valence specificity by vmPFC-tDCS. Findings comprise event-related magnetoencephalography (MEG) and functional magnetic resonance imaging (fMRI) data for passively viewed emotional natural scenes in two separate samples (Junghofer et al., 2017) as well as event-related MEG and behavioral data for emotional happy and fearful face processing (Winker et al., 2018). In all three studies, excitatory vmPFC-tDCS induced effects in neurophysiological correlates showing an increased activation during the processing of pleasant in comparison to unpleasant stimuli, while inhibitory vmPFC-tDCS led to the opposite pattern. These effects occurred in a broad network of regions, involved in visual emotional processing, e.g., ventral and dorsal pathways including occipital, parietal, and temporal cortical areas (Vuilleumier, 2005; Schupp et al., 2006). A conjoint analysis of MEG data of both studies across emotional scenes and faces revealed a consistent effect of relatively increased processing of pleasant stimuli after anodal/excitatory vmPFC-tDCS during late latency (~350–600 ms) spanning from right occipital and parietal cortex across the entire right temporal cortex (Winker et al., 2018). The latency and location of effects coincide with the late positive potential component (LPP), reflecting enhanced motivated attention to emotional relative to neutral stimuli (Cuthbert et al., 2000; Schupp et al., 2006). These results further support a valence specificity of vmPFC responsivity independent of the presented stimulus type. Moreover, a behavioral task—testing for tDCS effects on interpretation of ambiguous happy-fearful face morphs—yielded a significant influence of tDCS on participants’ emotional categorization. After excitatory vmPFC-tDCS, participants showed a relative positivity-bias by shifting the categorization of ambiguous faces toward pleasant expressions. Furthermore, for highly ambiguous faces, participants were faster in reacting to happier faces after excitatory stimulation, whereas after inhibitory stimulation a reversed effect with faster reactions to more fearful faces was reported. In addition to these findings from our group, other studies further support a general modulation capability of vmPFC by tDCS in the context of emotional processing paradigms (Chib et al., 2013; Mungee et al., 2014; Abend et al., 2018; Dittert et al., 2018; Van’t Wout et al., 2016). However, electrode placements in these studies differed from the one used in our experiments, which impedes comparability. We here applied the same stimulation protocol as in our previous studies to allow high comparability within this series of studies.

These encouraging results described above give reason to investigate excitatory vmPFC-tDCS as potential add-on therapy in psychiatric disorders that include emotional symptoms mentioned above. Among them, MDD classified as the “leading cause of disability worldwide” by the WHO^1^, is of specific interest. In the context of MDD, vmPFC-tDCS might have the potential to reduce a reported attentional bias away from pleasant and in favor of unpleasant stimuli (e.g., Everaert et al., 2012). To expand the previous results toward the investigation of stimuli more relevant in MDD, we here decided to replace fearful faces by sad faces as negative, unpleasant stimuli in all tasks of this study. Fearful faces are typically used to investigate attentional biases in anxiety disorders (e.g., Klahn et al., 2017) or high-anxious populations (e.g., Holmes et al., 2008; Moser et al., 2008), while for the investigation of attentional biases in mood disorders, sad faces are often applied (e.g., Gur et al., 1992; Gotlib et al., 2004; Surguladze et al., 2004; Joormann and Gotlib, 2007).

With the aim to (1) further replicate and generalize our previous findings to other stimulus material and to (2) study the relevance of vmPFC-tDCS within a prospective clinical MDD pilot study, we chose to investigate the impact of brain stimulation on the processing of happy and sad facial expressions in healthy control subjects. More precisely, we sought to reveal an attention increasing effect of excitatory compared to inhibitory stimulation on happy relative to sad face processing (i.e., a positivity bias). As in the preceding studies, we applied a passive viewing task with simultaneous MEG assessment as well as two behavioral tasks. The behavioral tasks included a face-morph and a dot-probe task that tested for interpretation and attention biases, respectively. All three tasks featured the presentation of facial stimuli with happy and sad expressions.

Consistent with previous results (Winker et al., 2018), we expected the influence of vmPFC-tDCS even to influence emotional ratings. Namely, for the face-morph task—in which participants were asked to categorize faces with morphed expressions on a sad-happy continuum—we expected interpretational shifts in emotional categorization specifically for highly ambiguous faces, while clearly expressive faces should not show any impact of brain stimulation on emotion ratings. Excitatory vmPFC stimulation should lead to an increased preferential categorization of ambiguous faces as happy faces (i.e., positivity bias in comparison to inhibitory stimulation). Further, reaction time analyses in the previous study delivered additional support for a valence-specific vmPFC-tDCS modulation. Therefore, under conditions of high ambiguity, we expected faster emotional categorization for happier faces after excitatory in comparison to inhibitory vmPFC-tDCS. In addition, we expected relatively faster reaction times for sadder faces after inhibitory in comparison to after excitatory vmPFC-tDCS. For the dot-probe task, we predicted an increased orientation toward happy faces compared to sad faces after excitatory compared to after inhibitory vmPFC-tDCS. Moreover, we expected an increased orientation to sad faces compared to happy faces after inhibitory compared to after excitatory vmPFC-tDCS. In addition, conjoint analyses for pleasant (happy) and unpleasant (sad, fearful) faces were conducted for all three tasks to test if a valence-specific modulation can be seen independent of the specific unpleasant emotion (sad, fearful).

For the MEG data, we hypothesized that excitatory vmPFC-tDCS should result in relatively increased neural evidence of motivated attention for happy vs. sad faces. Whereas for inhibitory vmPFC-tDCS, the opposite activation pattern should occur. We expected these effects—which were previously reported for emotional scenes (Junghofer et al., 2017) and faces (Winker et al., 2018)—to occur especially during late latency intervals (>300 ms) in occipito-temporal as well as parietal areas consistent with a modulated LPP component. Furthermore, as identified in our prior stimulation studies, we expected differential activity also in early (0–100 ms) and mid-latency time intervals (100–300 ms), as involvement of prefrontal cortex in visual emotional processing begins soon after stimulus processing and remains active across time (for reviews, see Vuilleumier, 2005; Pessoa and Adolphs, 2010).

## Materials and Methods

### Participants

Recruitment took place in Muenster, Germany, *via* flyers and email newsletters of the University. In total, we assessed 41 participants of whom 40 participants (30 female) were included in the initial analyses. One participant had to be excluded beforehand due to an increased amount of bad trials in the MEG data and identification as outlier in both behavioral tests. Additionally, one participant—identified as outlier—was excluded in the dot-probe task and two further participants were excluded from the MEG analysis due to technical issues. Before the start of the experimental session, participants gave written informed consent in accordance with the Declaration of Helsinki, approved by the University’s Human Subjects Review Board. For every participant, we ensured that there were no current or past severe neurological disorders and no history of psychiatric disorders. Furthermore, we assessed the Beck Depression Inventory II (BDI II; Beck et al., 1996) and the State Trait Anxiety Inventory (STAI; Laux et al., 1981) at the beginning of the experimental session. In the end, participants filled in the Symptom Check List (SCL-90-S; Franke, 2014). Demographic data and results of the questionnaires from participants of the current and the previous study (Winker et al., 2018) are displayed in Table 1.

**Table 1:** To ensure the assessment of healthy samples, clinical questionnaires with regard to symptoms of major depressive disorder (MDD; BDI-II) as well as general psychiatric symptoms (SCL-90-S) were assessed in the current study (Happy/Sad).

	Happy/Sad *N* = 40 M (SD)	Happy/Fear *N* = 40 M (SD)
**Age**	23.8 (2.68)	24.6 (2.91)
**STAI**		
State	33.48 (4.99)	30.15 (5.02)
Trait	32.3 (5.03)	30.37 (6.58)
Trait *T*-value	47.77 (5.52)	44.9 (8.83)
**BDI-II**	2.88 (2.49)	2.2 (2.57)
**SCL-90-S**		
GSI	40 unremarkable	40 unremarkable
PSDI	40 unremarkable	40 unremarkable
PST	15.03 (8.86)	10.1 (6.79)

*To check for conspicuities regarding symptoms of trait- and state-dependent anxiety, the STAI was assessed. Due to conjoint analyses across independent samples, the assessed data from the previous study (Happy/Fear) is reported here in addition. There were no indications for conspicuities in the assessed samples. STAI, State Trait Anxiety Inventory; BDI, Beck Depression Inventory; SCL, Symptom Check List; GSI, Global Severity Index; PSDI, Positive Symptom Distress Index; PST, Positive Symptom Total*.

### tDCS

For the tDCS, we used a DC Stimulator Plus (NeuroConn, Ilmenau, Germany). The stimulation protocol (similar to Junghofer et al., 2017; Winker et al., 2018) was as follows: we applied 1.5 mA of current strength for a duration of 10 min *via* two electrodes coated in saline-soaked sponges. One electrode (3 × 3 cm)—serving as a stimulating component—was positioned above the forehead at 10–20 electrode position Fp. The other (5 × 5 cm) was positioned centrally below the chin and served as an extracephalic reference electrode. This kind of stimulation circumvents the inherent reference problem of tDCS (i.e., simultaneous inhibitory neuronal stimulation under cathodal and excitatory stimulation under anodal electrode) and can thus be termed “quasi” reference free. All participants received two types of stimulation: excitatory (anode above forehead—cathode below chin) and inhibitory stimulation (cathode above forehead—anode below chin). Order of stimulation (excitatory first/inhibitory second or inhibitory first/excitatory second) was balanced across participants. Participants were blind with regard to the different stimulation conditions.

### Stimuli

The stimuli we used in the study comprised of posed facial expressions with neutral, sad, and happy emotions from a frontal view. Photographs were taken from the Radboud Faces Database (Langner et al., 2010), NimStim Set of Facial Expressions (Tottenham et al., 2009), and the Karolinska Directed Emotional Faces Database (Lundqvist et al., 1998).

For the passive viewing task (MEG measurement), we presented facial stimuli from 64 different individuals with either happy (32) or sad expression (32). The face-morph task featured four face pairs of different individuals containing a happy and a sad facial expression of the same individual, respectively, which were morphed into each other (see “Face-Morph Task” section for a detailed description). The dot-probe task featured 60 different face stimuli of 20 individuals in total. From every individual, happy, sad, and neutral expressions were used in the presentation of emotional-neutral face pairs (see “Dot-Probe Task” section for a detailed description). For the MEG measurement and each behavioral task, different subsets of faces were used.

We chose the final set of stimuli for all three tasks based on ratings for happiness, sadness, and authenticity of the facial expression, which were assessed in a pilot study before in independent samples of four individuals. Furthermore, for all subsets stimulus brightness and contrast did not differ statistically between conditions. In all tasks, conditions were balanced for gender.

### Procedure

**Figure 1:**
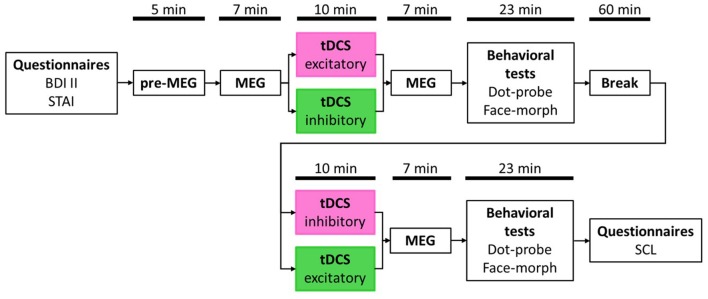
Study timetable. Order of stimulation was balanced with half the participants receiving excitatory transcranial direct current stimulation (tDCS) first and inhibitory tDCS second (pink box) and vice versa for the other half (green box). Magnetoencephalography (MEG) and behavioral test durations are presented as average values, respectively, due to jittered inter-trial intervals (ITIs) in the passive viewing task (MEG) and individual differences in the behavioral tasks.

After filling in the informed consent form and both, the BDI II and the STAI questionnaires (see Figure 1 for an overview of the procedure), participants were prepared for the MEG data acquisition. Beforehand, the head shape of each participant was registered with a 3Space Fastrak (Polhemus, Colchester, VT, USA) localization system. We then asked participants to take place in the MEG scanner. Preceding the first MEG measurement (pre-MEG), participants viewed all facial stimuli in a randomized order twice to get accustomed to the stimulus material. In succession, they received an instruction on screen to relax and sit still during the time of measurement and to view all randomly presented facial stimuli, three times each, attentively. An MEG measurement run took on average 7 min. Afterward, participants received the first of two tDCS outside the MEG scanner. With the end of stimulation, they immediately returned into the MEG chamber for the next measurement run. All MEG measurement runs were similar in length and presentation of stimuli. However, for each run, stimuli were presented in a new randomized order. Subsequent to the first post-tDCS MEG run, participants accomplished both behavioral tests, the dot-probe- and the face-morph task, again outside of the MEG scanner. To dissipate residual tDCS aftereffects (Nitsche et al., 2005), participants waited for an additional amount of time leading to approximately 90 min between the end of the first and the start of the second tDCS session. After the second tDCS session, participants were seated again in the MEG scanner for the second post-tDCS MEG measurement again followed by both behavioral tasks. Order of the behavioral tasks was balanced across participants with half of the participants starting both behavioral test sessions with the dot-probe task and the other half with the face-morph task.

### Behavioral Tests

Behavioral testing was conducted in computerized form. As input device participants used a DirectIN PCB keyboard (Empirisoft) to reduce reaction time jittering caused by hardware to a minimum (<1 ms). For visualization, we used an 85 Hz G90fB CRT monitor (ViewSonic, Brea, CA, USA). Statistical analysis of all behavioral data, if not stated otherwise, was conducted with SPSS (IBM).

#### Face-Morph Task

**Figure 2:**
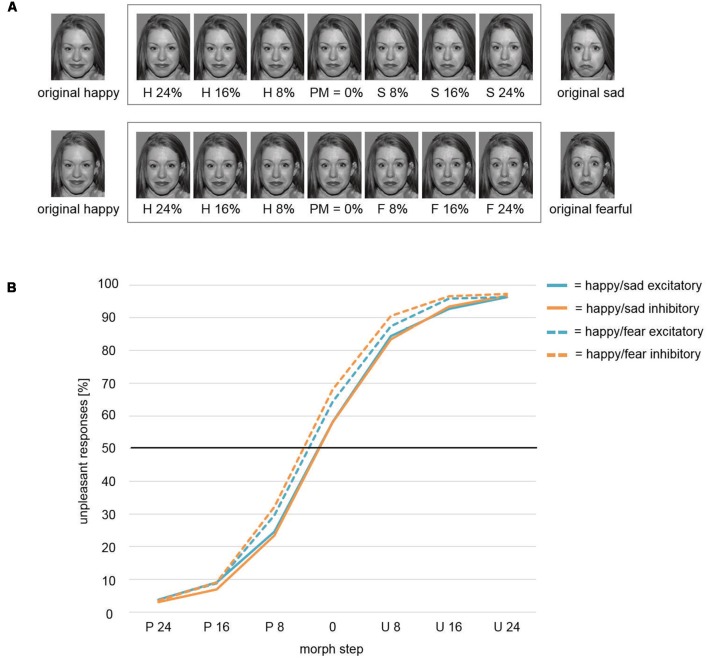
**(A)** Face-morph task. Morphed facial stimuli from left to right show increasing sad (top row)/fearful (bottom row) and decreasing happy facial expressions. The pre-assessed perceptual midpoint (PM) is centrally positioned. Six morph steps around PM were chosen for optimal response function coverage. Letters indicate the relatively dominant emotion (H, happy; S, sad; F, fearful). Numbers within the frame indicate the added percentages toward the respective emotion (e.g., H 16% = PM + 16% increase in happy expression/16% decrease in sad/fearful expression). Stimuli outside the frame were used for creating face morphs and were not featured in the respective final tasks. To avoid morphing artifacts, stimuli were prepared by means of Photoshop software (Adobe Systems) beforehand. Therefore, the here exemplarily displayed happy faces differed slightly across studies. However, retouching only focused on the hair area. No changes were applied to the facial area. The identifiable image is model #1 from the NimStim Set of Facial Expressions (Tottenham et al., 2009) and is released for publication in scientific journals. **(B)** Weibull fitted response function. The Weibull function modeled mPM and the shape parameter β did not differentiate between stimulation conditions Inhibitory and Excitatory for the presentation of ambiguous happy-sad face morphs. However, a significant main effect for factor Study revealed an overall shift of the mPM for happy-sad faces toward happier faces relative to happy-fearful faces. Thus, participants in study Happy/Sad needed higher amounts of unpleasant, i.e., sad facial expressions to categorize an ambiguous face morph as unpleasant/sad in comparison to participants in study Happy/Fear. P, Pleasant; U, Unpleasant.

We conducted the face-morph task (based on McMahon and Leopold, 2012) as described by Winker et al. (2018) to investigate the emotional interpretation of ambiguous emotional expressions (happy vs. sad). To create ambiguous face stimuli, we morphed happy and sad faces together by means of PsychoMorph software^2^ (Tiddeman and Perrett, 2002). This procedure led to a continuum of facial expressions from the original happy (100% happy, 0% sad) to the original sad face stimulus (0% happy, 100% sad) in 101 steps. Out of this continuum, we chose morph steps with different amounts of ambiguity for the final task. In a pre-test with eight participants, who were not considered in the sample of this study, we first assessed the most ambiguous facial stimulus per stimulus set. This stimulus is defined as the perceptual midpoint (PM) of this continuum with mean categorizations of 50% happy and 50% sad. The PM-stimulus as well as six neighboring ambiguous stimuli in morph steps of PM ± 8%, PM ± 16%, and PM ± 24% (i.e., seven stimuli per face pair) were used for the task (Figure 2A, top row). These morph steps enabled a gradient from fairly distinct sad and happy facial expression to maximal ambiguity. For the face-morph task, participants were instructed to categorize presented faces in a forced-choice procedure as sad or happy face, respectively. In the context of tDCS, we expected a modulation effect on the PM revealing itself in a relative difference between post-excitatory and post-inhibitory stimulation. We thus termed this outcome measure modulated perceptual midpoint (mPM). An mPM shifted relatively toward happy faces would, for instance, indicate an induced interpretational shift with the identification of ambiguous faces as more happy and/or less sad (i.e., reduced percentage of ratings as unpleasant in Figure 2).

The task began with two preparation blocks to allow participants to get accustomed to the task. Then the task block followed. Here, we presented 112 trials with repetition of the seven morph steps for 16 times each. Order of morph steps was randomized but each morph step was shown only once before the next repetition of all seven morph steps would start again. We instructed participants to categorize the presented stimulus as fast and correct as possible. Furthermore, they were told to categorize faces intuitively. Especially those faces, which were difficult to assign to one of both emotions. Responses were given by both index fingers, which rested on two keyboard buttons throughout the whole task. Each trial started with the presentation of a white fixation cross for 500 ms followed by the presentation of a morph stimulus. Presentation time of the stimulus was either terminated by a button press or after 2,000 ms. Absence of a button press during a trial counted as “miss.” This procedure (two preparation blocks, one task block) was similar for all four morphed stimulus sets. The face-morph task was written and executed with the Psychophysics Toolbox software package (Brainard, 1997) in Matlab (The MathWorks, Natick, MA, USA).

In preparation for statistical analysis, we excluded all “miss” trials. All remaining trials were used for a fit calculation of a Weibull cumulative distribution function ($F(x)=1-e^{-{\left(\alpha x\right)}^{\beta }}$) with parameters *α* (scale parameter) and *β* (shape parameter). A fit was calculated per participant across all four face pairs. Preprocessing and the Weibull fit were executed in Matlab. The calculated output additionally comprised a parameter expressing the goodness of fit (GoF; for results see Supplementary Material). We conducted *t*-tests or Wilcoxon signed-rank tests, dependent on the underlying sample distribution, for the mPM, β, and the GoF parameter between both post-tDCS conditions (Excitatory, Inhibitory). Moreover, we conducted a repeated measures analysis of variance (rmANOVA) for the dependent variable Reaction Time and the factors Morph Step (H 24%, H 16%, H 8%, 0% (mPM), S 8%, S 16%, S 24%; H: Happy, S: Sad) and Stimulation (Excitatory, Inhibitory). For this analysis, we additionally excluded single trial outliers with reaction times > 3 × standard deviation (SD). SDs were based on the average reaction time per participant.

#### Dot-Probe Task

To investigate attentional orienting toward or away from happy and sad facial stimuli in the light of tDCS modulation, we conducted a dot-probe task adapted from MacLeod et al. (1986). The task was to locate a dot presented either on the left or on the right of a computer screen *via* button press. Preceding the dot, a pair of an emotional and a neutral face from the same individual was presented at the possible left and right-sided dot locations. For emotional stimuli, sad faces and happy faces were used. As we were interested in the influence of the emotional cues, we defined a dot appearing at the position of a happy or sad face as congruent and as incongruent if it appeared at the other of both locations, where the neutral cue would be presented. A decelerated or accelerated reaction time between conditions could then reveal information about the focus of attention. Therefore, we calculated the attentional bias (MacLeod and Mathews, 1988). The attentional bias describes in a single value the relation between reaction times for congruent and incongruent trials. It is calculated as follows: $\frac{\left(RdLe-RdRe)+(LdRe-LdLe\right)}{2}$ with R = right, L = left, d = dot, e = emotional face. A positive value of the attentional bias here describes a faster reaction to congruent stimuli in comparison to incongruent stimuli and vice versa for a negative value. Thus, a positive value can be interpreted here as an orienting toward the emotional and away from the neutral prime, whereas a negative value would indicate an orienting toward the neutral and away from the emotional prime.

Participants were instructed to identify the location of the presented dot (left or right) and react as fast and correctly as possible. Their index fingers both rested on a left and right button of the keyboard, respectively. A trial began with the presentation of a white fixation cross in the center of the screen for 1,000 ms. Then, presentation of an emotional-neutral stimulus pair followed with a duration of 1,000 ms. In direct succession, a white dot appeared on one side of the screen. The task consisted of 160 trials in total, with 80 happy-neutral and 80 sad-neutral face pairs. For half of the respective face pairs, the dot appeared at the location of an emotional stimulus (congruent) and for the other half, it appeared at the location of a neutral stimulus (incongruent). Left and right side appearances were balanced within each face-pair. The dot-probe task-script was written and run with Presentation software (Neurobehavioral Systems).

For the dot-probe task, preprocessing of the reaction time data consisted of removing all trials with a wrong button press (error trials) as well as trials with reaction times <100 ms and >3,000 ms. In addition, we excluded all trials with reaction times > 3 × SD per participant. An rmANOVA with dependent variable Attentional Bias and factors Valence (Happy, Sad) and Stimulation (Excitatory, Inhibitory) was calculated.

#### Behavioral Data Analysis for Pleasant and Unpleasant Faces in General

As we were also interested in commonalities of valence processing across different stimulus types, we conducted conjoint analyses across the current study (Happy/Sad) and the previous study, which featured the same paradigm but presented fearful faces instead of sad faces (Happy/Fear; Winker et al., 2018). Here, we applied a mixed-model ANOVA (mmANOVA) with within-subjects factors Valence (Pleasant, Unpleasant), Stimulation (Excitatory, Inhibitory), and between-subjects factor Study (Happy/Sad, Happy/Fear) for the different dependent variables (dot-probe task: Attentional Bias; face-morph task: GoF, *β*, mPM, Reaction Time) if statistically legitimate. Consequentially, factor level Pleasant included reactions to happy faces and Unpleasant combined trials of fearful and sad faces. For an example, see Figure 2A on the face-morph task and compare top row (happy-sad face morphs) with bottom row (happy-fearful face morphs).

### MEG Measurement and Analysis

During the passive viewing task, every facial stimulus was presented for 600 ms with a jittered inter-trial interval (ITI) of 1,000–2,000 ms. Face stimuli were positioned with their nasion centrally on a medium gray background and were presented with a vertical visual angle of 8.4° and varying horizontal visual angles due to natural differences across stimuli. ITIs featured a white fixation cross positioned at the center of the screen. A measurement run contained 192 trials with a total presentation of each stimulus threefold. Every stimulus was displayed once before stimulus presentation repeated in a new randomized order. We controlled for transition probabilities across all possible order alignments between trials. Furthermore, we applied repetition restrictions, which allowed stimuli of one condition (happy, fearful) to appear in a row for a maximum of four consecutive trials.

We conducted MEG measurements with a 275 whole-head sensor system (CTF Systems) with first-order axial gradiometers. To control for movement and head position within the MEG sensor cap, we assessed head coordinates by means of landmark coils positioned on the nasion and in both earlobes. We measured data with a sampling rate of 600 Hz to prevent anti-aliasing effects in a frequency band of 0–150 Hz. After measurement, data were down-sampled to 300 Hz and we applied a 0.1 Hz high-pass filter (zero-phase second-order Butterworth) as well as a 48 Hz low-pass filter (zero-phase fourth-order Butterworth). In a next step, we prepared trials by splitting the signal into 800 ms epochs ranging from −200 ms before stimulus onset to 600 ms after stimulus onset. Trials were baseline-adjusted by subtraction of the last 150 ms before stimulus onset (−150 to 0 ms). The resulting trials were then averaged into cell means of participants × MEG runs × valence conditions. Those files contained mean activation maps across each time point and sensor position. We then calculated the corresponding source maps *via* Minimum-Norm estimation (L2-MNE; Hämäläinen and Ilmoniemi, 1994). A spherical shell with evenly distributed 2 × 350 dipoles served as source model. Topographies of the L2-MNE were established with a Tikhonov regularization parameter of *k* = 0.1.

Statistical analysis comprised ANOVAs for the L2-MNE data. To prevent false-positive results due to α error inflation, we applied a non-parametric cluster permutation (for a detailed description of the method see Maris and Oostenveld, 2007). With this procedure, we tested spatio-temporal clusters of an effect of interest against a distribution of 1,000 random permutations of the given data by means of a predefined α-level. In order to add an effect to a cluster, the respective spatio-temporal point had to surpass a *p*-value of 0.05 (sensor-level criterion). Subsequently, all effects falling below this threshold were added up to a so-called cluster mass (CM; in this case summation of all *F*-values) and tested against the randomly permuted data. If the cluster mass reached a *p*-value < 0.01 in comparison to the biggest cluster mass of each of the 1,000 permutations (cluster-level criterion), it surpassed the critical cluster mass (CCM) and thus was considered significant. We conducted cluster mass-analyses for predefined intervals of 0–100 ms, 100–200 ms, and 300–600 ms to allow for comparability with previous studies (Junghofer et al., 2017; Winker et al., 2018). In the case of clusters reaching the starting or ending border of an interval, the interval was extended for 50 ms in the respective temporal direction and reanalyzed. This procedure allowed an estimation of the temporal onset and offset of the already significant cluster independent of the* a priori* defined intervals. However, novel clusters appearing during this *post hoc*-reanalysis were not taken into consideration. In light of our hypothesis, we initially ran cluster mass-analyses for the interaction effect Stimulation (Excitatory, Inhibitory) × Valence (Happy, Sad). Furthermore, to investigate a general stimulation effect on pleasant and unpleasant valence, we conducted an additional analysis across the data sets of the current study (Happy/Sad) and our previous study (Happy/Fear; Winker et al., 2018). Here, a conjoint cluster mass-analysis for a two-way interaction Stimulation × Valence (Pleasant, Unpleasant), with Unpleasant consisting of sad and fearful face trials, was investigated. However, to scan for differences between stimulus categories and/or data sets, we additionally ran an analysis for the three-way interaction Stimulation × Valence × Study (Happy/Sad, Happy/Fear). In case of a significant three-way interaction effect, *post hoc*-ANOVAs were run separately for both study samples to identify the underlying driving factor of the effect. Preprocessing and analysis of the MEG data was conducted using EMEGS software^3^ (Peyk et al., 2011).

## Results

For all ANOVAs Greenhouse-Geisser corrections were applied in cases of violation of sphericity.

### Behavioral Tests

#### Face-Morph Task

##### Modulated Perceptual Midpoint (mPM) and Shape Parameter β: Happy and Sad Faces

For the mPM, the *t*-test between conditions Excitatory and Inhibitory resulted in a non-significant difference (*t*_(39)_ = 0.7, *p* = 0.488). The comparison of shape parameter β after excitatory and inhibitory stimulation yielded no significant difference (*t*_(39)_ = 0.02, *p* = 0.986) as well (solid lines; Figure 2B).

##### Modulated Perceptual Midpoint (mPM) and Shape Parameter β: Pleasant and Unpleasant Faces

In an mmANOVA with dependent variable mPM and factors Stimulation and Study, the main effect Stimulation did not show significant differences between conditions (*F*_(1,75)_ = 1.63, *p* = 0.206; Figure 2B). A main effect Study (*F*_(1,75)_ = 7.46, *p* = 0.008) was driven by the fact that the mPM for the study Happy/Sad (solid lines) was overall shifted toward more pleasant categorizations in relation to the study Happy/Fear (dashed lines). A significant interaction Stimulation × Study (*F*_(1,75)_ = 5.3, *p* = 0.024) illustrates the difference of the impact of stimulation between both studies, with distinct stimulation effects of Excitatory and Inhibitory in the Happy/Fear but clearly no effects in the Happy/Sad study.

The sample of study Happy/Fear remained non-normally distributed for parameter β, therefore we ran a Wilcoxon signed-rank test across all sample pairs, leading to a non-significant difference between conditions Excitatory and Inhibitory (*Z* = −0.75, *p* = 0.456).

##### Reaction Time: Happy and Sad Faces

**Figure 3:**
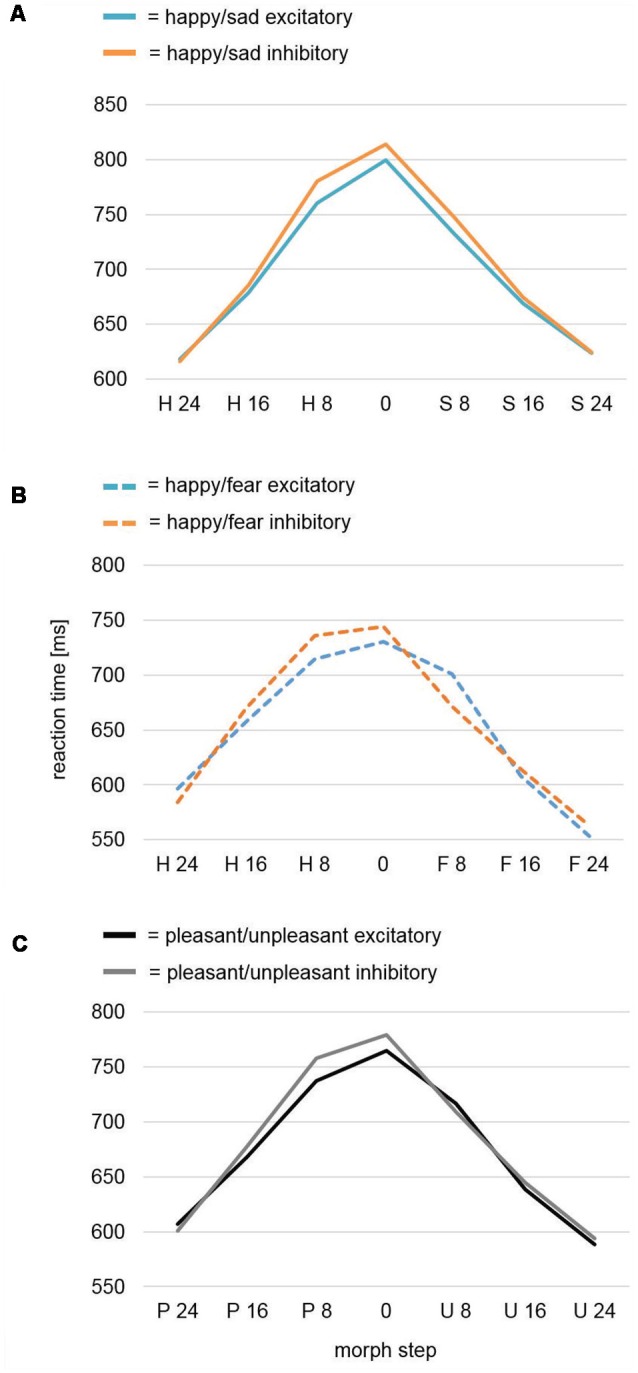
Reaction time analysis of the face-morph task data. **(A)** Reaction time analysis for study Happy/Sad. After excitatory vmPFC-tDCS, participants were faster for highly ambiguous faces in comparison to after inhibitory vmPFC-tDCS as revealed by a significant quadratic contrast. No difference was found between categorization of happier and sadder face morphs. **(B)** Reaction time analysis for study Happy/Fear. In a previously assessed sample featuring happy-fearful face morphs (Winker et al., 2018), a cubic trend for analysis of all morph steps and a significant cubic contrast after exclusion of the outer (most distinct) morph steps occurred. In contrast to happy-sad face morphs, excitatory vmPFC-tDCS might have specifically decreased reaction times for happier, highly ambiguous face morphs, whereas inhibitory vmPFC-tDCS decreased reaction times for more fearful, highly ambiguous face morphs. **(C)** Conjoint reaction time analysis Pleasant/Unpleasant. An analysis across both studies (Happy/Sad, Happy/Fear) again featured a significant cubic contrast for Morph Step in the interaction Stimulation × Morph Step. Upon visual inspection, more pleasant (happy), highly ambiguous face morphs seemed to consistently show a decreased reaction time after excitatory vmPFC-tDCS, whereas more unpleasant (sad, fearful), highly ambiguous face morphs did not show any difference.

An rmANOVA for dependent variable Reaction Time revealed a significant main effect for the factor Morph Step (*F*_(6,234)_ = 75.73, *p* < 0.001) with higher ambiguity in face-morph stimuli leading to slower reaction times (Figure 3A). While the factor Stimulation and the interaction of Stimulation × Morph Step showed no effects (*F*_(1,39)_ = 0.39, *p* = 0.535; *F*_(6,234)_ = 1.32, *p* = 0.266), the quadratic contrast of factor Morph Step in the interaction Stimulation × Morph Step yielded a significant effect (*F*_(1,39)_ = 4.38, *p* = 0.043). This effect was driven by a difference between Stimulation conditions with faster reaction times for the most ambiguous faces after excitatory compared to after inhibitory stimulation, while stimulation had no effects on categorizations of the easier identifiable happy or sad expressions.

##### Reaction Time: Pleasant and Unpleasant Faces

We further conducted an mmANOVA across studies (for results of the previous study Happy/Fear alone, see Figure 3B) with dependent variable Reaction Time and factors Morph Step, Stimulation, and Study (Figure 3C). Of course, the main effect Morph Step was highly significant (*F*_(6,468)_ = 121.23, *p* < 0.001). The main effect Study revealed a trend (*F*_(1,78)_ = 3.62, *p* = 0.061) reflecting overall longer reaction times—i.e., harder differentiation—in the Happy/Sad than in the Happy/Fear study. The interaction Morph Step × Study also revealed a trend (*F*_(6,468)_ = 2.37, *p* = 0.072). The main effect of Stimulation and all interactions with the factor Stimulation (Stimulation × Study, Morph Step × Stimulation, Morph Step × Stimulation × Study) remained non-significant with* p*-values > 0.2. However, within-subjects contrasts revealed a significant cubic effect for Morph Step in the interaction Morph Step × Stimulation (*F*_(1,78)_ = 4.15, *p* = 0.045). Upon visual inspection, the direction of effect showed faster reaction times after excitatory compared to after inhibitory stimulation for highly ambiguous face morphs with higher amounts of pleasant (happy) emotion. Distinct morphs, as well as highly ambiguous face morphs with higher amounts of unpleasant (fearful, sad) emotion in contrast, showed no differences between stimulations (Figure 3C).

#### Dot-Probe Task

##### Attentional Bias: Happy and Sad Faces

**Figure 4:**
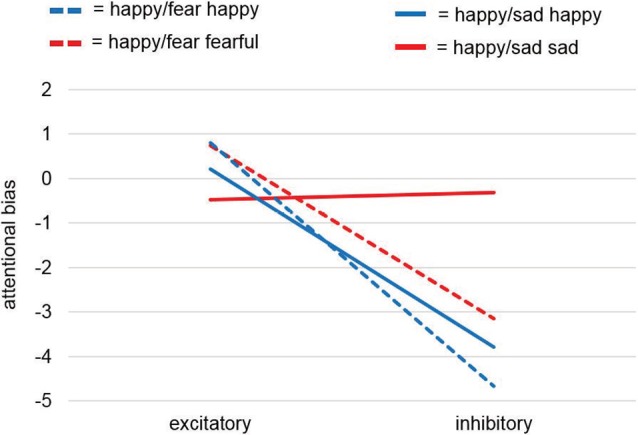
Attentional bias analysis of the dot-probe task. Analyses of the attentional bias (for calculation see “Dot-Probe Task” section) for study samples Happy/Sad and Happy/Fear together revealed a significant main effect for factor Stimulation and thus pointed to a consistent effect for happy faces with excitatory vmPFC-tDCS compared to inhibitory vmPFC-tDCS leading to a relatively positive bias, i.e., a faster reaction to a dot after the presentation of a relevant cue (here, happy faces) at the same location. This was confirmed by a separate analysis for happy faces only yielding a significant difference between stimulations. For unpleasant faces only (sad, fearful) no differences were found.

rmANOVA results for dependent variable Attentional Bias yielded no significant effects neither for factors Stimulation and Valence (*F*_(1,38)_ = 0.96, *p* = 0.334; *F*_(1,38)_ = 0.28, *p* = 0.602) nor for the interaction Stimulation × Valence (*F*_(1,38)_ = 1.03, *p* = 0.317; Figure 4).

##### Attentional Bias: Pleasant and Unpleasant Faces

While the dot-probe task in the preceding Happy/Fear study also revealed non-significant results, the conjoint analysis across both studies, in fact, revealed a significant main effect of Stimulation (*F*_(1,75)_ = 4.22, *p* = 0.044). Across both studies, excitatory stimulation led to a positive attentional bias compared to an overall more negative attentional bias after inhibitory stimulation. All other comparisons (Valence, Study, Stimulation × Study, Stimulation × Valence, Valence × Study, Stimulation × Valence × Study) remained non-significant with *p*-values > 0.2.

Visual inspection of the results showed comparable effects of attentional biases toward happy faces after excitatory compared to after inhibitory stimulation in both studies, whereas stimulation induced biases for sad faces and fearful faces differed (Figure 4). To investigate these differential effects further, we analyzed in two *post hoc*-ANOVAs split up for pleasant and unpleasant valence if this effect was driven by happy faces. The *post hoc*-mmANOVA for happy faces only resulted in a significant main effect for factor Stimulation (*F*_(1,75)_ = 5.04, *p* = 0.028). For main effect Study and interaction Stimulation × Study, results were non-significant (*F*_(1,75)_ < 0.01, *p* = 0.951; *F*_(1,75)_ = 0.12, *p* = 0.727). In contrast, no significant results were found for unpleasant faces only (Stimulation: *F*_(1,75)_ = 0.81, *p* = 0.37; Study: *F*_(1,75)_ = 0.2, *p* = 0.656; Stimulation × Study: *F*_(1,75)_ = 0.97, *p* = 0.327). Overall, these results point to a stimulation effect specifically for pleasant material, namely happy faces, consistent with our hypothesis.

### MEG

The analysis of the neurophysiological data revealed significant interactions with the factor stimulation in all investigated time intervals (0–100 ms, 100–200 ms, 200–300 ms, and 300–600 ms).

#### Interaction Stimulation × Valence: Happy and Sad Faces

**Figure 5:**
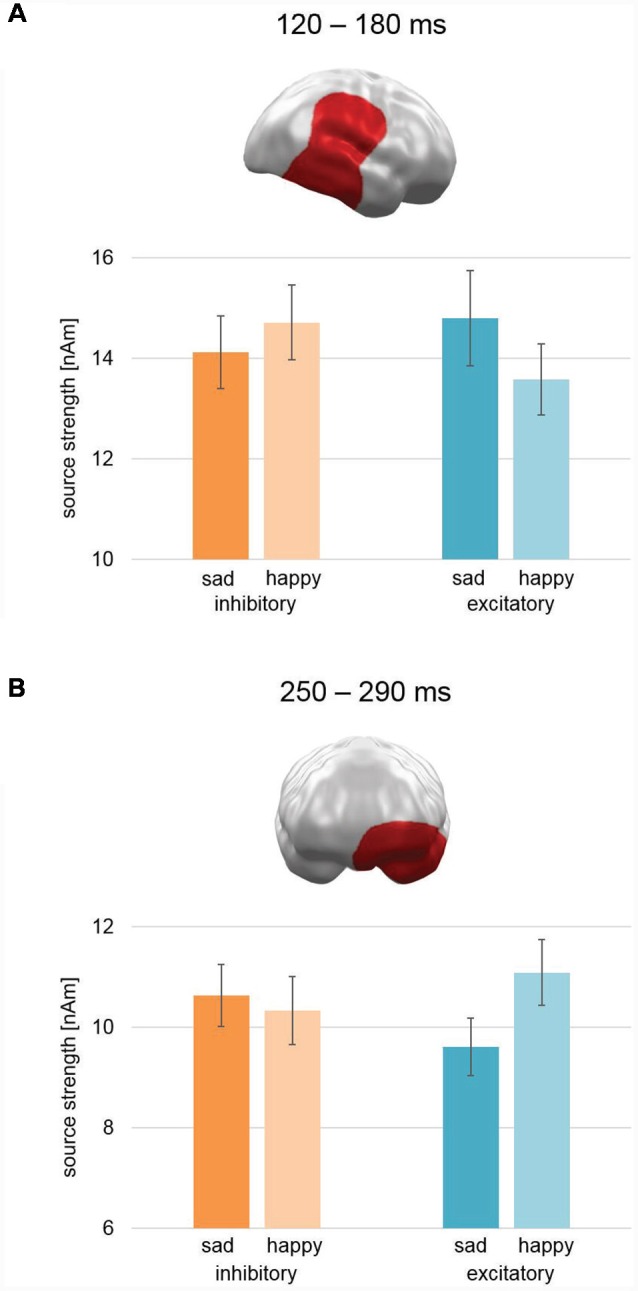
Spatio-temporal clusters with significant interactions of Stimulation × Valence. **(A)** An early to mid-latency cluster spanning right temporal, parietal, and frontal areas—including temporal parietal junction and superior temporal cortex—showed an activation pattern opposite to our hypothesis: after excitatory vmPFC-tDCS, sad faces showed relatively higher activation than happy faces and vice versa after inhibitory vmPFC-tDCS. **(B)** A mid-latency cluster at left orbitofrontal and temporal pole areas revealed an effect consistent with our hypothesis: after excitatory vmPFC-tDCS, happy faces showed increased activation in comparison to sad faces and vice versa after inhibitory vmPFC-tDCS. Bar graph insets indicate standard error of the mean (± SEM).

A cluster was found in the early mid-latency interval (100–200 ms) between 120–180 ms after picture onset (CCM [990th biggest cluster mass of 1,000 permutations; *p* < 0.01], CCM: 572; Cluster Mass with correct labeling: CM: 1299.2; Figure 5A). It appeared at right temporal, parietal, and frontal areas—covering the temporo-parietal junction (TPJ) and STC—and yielded a pattern showing relatively increased activation for sad faces in comparison to happy faces after excitatory stimulation, whereas after inhibitory stimulation happy faces were processed stronger than sad faces. This pattern was reversed to our hypothesis of a modulation of pleasant valence.

In the later mid-latency time interval (200–300 ms), the cluster permutation test revealed a spatio-temporal cluster consistent with our hypothesis between 250 and 290 ms with a significant interaction of Stimulation × Valence (CCM: 757.5; CM: 892.2; Figure 5B). This cluster spanned over left orbitofrontal cortex (OFC) and temporal pole and revealed relatively stronger activation for happy faces compared to sad faces after excitatory stimulation and vice versa after inhibitory stimulation. Thus, the earlier right temporo-parietal cluster and the later left hemispheric prefrontal cluster revealed different patterns with respect to the modulation of valence biases for happy vs. sad faces.

#### Interaction Stimulation × Valence × Study: Pleasant and Unpleasant Faces

**Figure 6:**
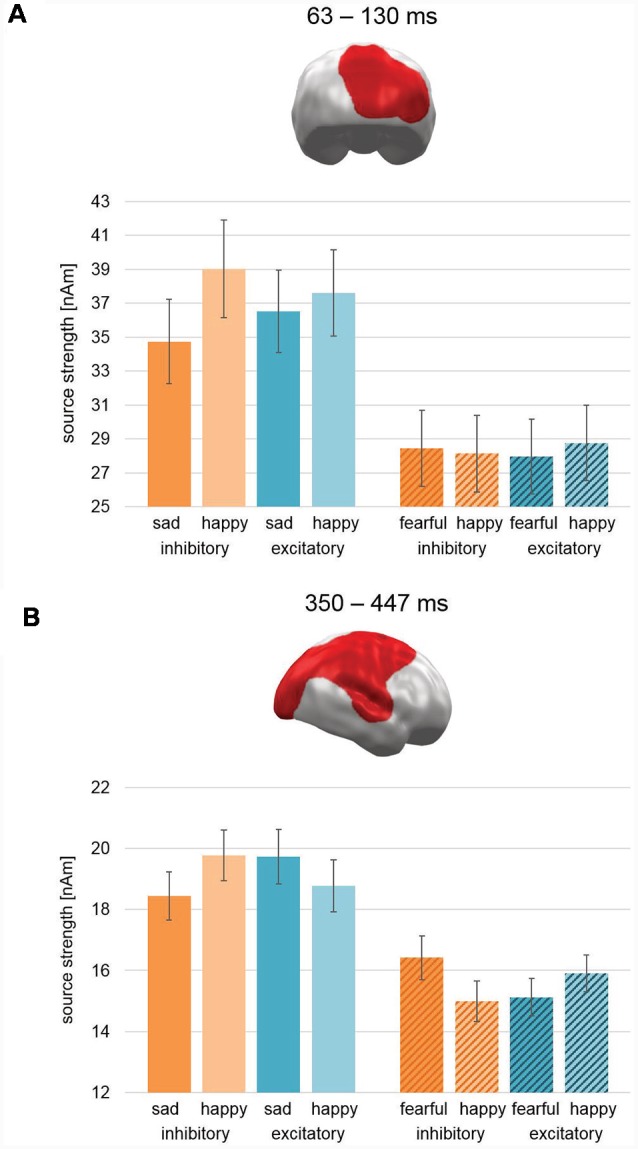
Spatio-temporal clusters with significant three-way interactions of Stimulation × Valence × Study. **(A)** An early cluster at right occipital cortex regions revealed an effect consistent with our hypothesis for the Happy/Fear study with happy faces showing increased activation compared to fearful faces after excitatory vmPFC-tDCS and vice versa after inhibitory vmPFC-tDCS. For the Happy/Sad study, happy faces showed after both stimulations a relatively higher activation than sad faces, but this difference was relatively smaller after excitatory compared to inhibitory vmPFC-tDCS. **(B)** Similar to the early latency cluster, the activation pattern in a large late cluster spanning right occipital, parietal, and frontal, as well as superior temporal cortex for the Happy/Fear study was consistent with our hypothesis. However, an exact opposite activation pattern and thus contrary to our hypothesis occurred for study Happy/Sad. It is noteworthy that the spatio-temporal cluster reflects the finding of a two-way interaction consistent with our hypothesis previously reported for Happy/Fear alone (Winker et al., 2018). Bar graph insets indicate standard error of the mean (± SEM).

Cluster permutation analyses across both studies revealed no clusters with significant interactions of Stimulation × Valence in any time interval. However, two clusters revealed a three-way Stimulation (Excitatory, Inhibitory) × Valence (Pleasant, Unpleasant) × Study (Happy/Sad, Happy/Fear) interaction: the first cluster emerged during early latencies in right occipital areas (63–130 ms; CCM 637; CM: 790.3; Figure 6A). For study Happy/Sad, it featured an interaction effect inconsistent with our* a priori* hypothesis (Excitatory: Sad < Happy, Inhibitory: Happy > Sad) showing an overall higher activation for happy faces relative to sad faces, while the difference between happy and sad faces decreased from Inhibitory to Excitatory (Figure 6A, plain bars). For study Happy/Fear, data showed stronger activation for happy faces in comparison to fearful faces after excitatory stimulation and vice versa after inhibitory stimulation and thus the pattern predicted for happy vs. fearful faces (Figure 6A, striped bars). *Post hoc*-ANOVAs revealed significant two-way interaction effects for both data sets, respectively (Happy/Sad: *F*_(1,37)_ = 22.88, *p* < 0.001; Happy/Fear: *F*_(1,38)_ = 5.9, *p* = 0.02).

During late-latencies, a second right hemispheric cluster revealed a significant three-way interaction (350–447 ms; CCM: 1709.5; CM 3219.5; Figure 6B). Covering widely distributed occipital, parietal, and frontal areas as well as TPJ, again it featured an activation pattern with Happy/Sad showing an interaction effect reversed to our hypothesis (i.e., increased activation for sad faces relative to happy faces after excitatory stimulation and vice versa after inhibitory stimulation; Figure 6B, plain bars). For study Happy/Fear, the pattern in this cluster showed stronger activation for happy faces in comparison to fearful faces after excitatory stimulation and vice versa after inhibitory stimulation yielding again the pattern hypothesized for happy vs. fearful faces (Figure 6B, striped bars). Again, *post hoc*-ANOVAs revealed significant two-way interactions for both study samples (Happy/Sad: *F*_(1,37)_ = 11.37, *p* = 0.002; Happy/Fear: *F*_(1,38)_ = 34.33, *p* < 0.001).

Therefore, in the combined analysis of Happy/Fear and Happy/Sad data, both clusters showed an interaction Stimulation × Valence supporting the hypothesis of negative valence increase (relative to happy faces) after inhibitory compared to excitatory stimulation for fearful faces but the opposite for sad faces.

## Discussion

Three previous studies in our lab revealed convergent neuronal and behavioral evidence that excitatory tDCS of the vmPFC resulted in relatively enhanced neural processing of pleasant material while inhibitory stimulation increased the processing of unpleasant emotional stimuli. While the prior functional magnetic resonance imaging (fMRI) and MEG studies compared the impact of stimulation on pleasant and unpleasant emotional scene perception (Junghofer et al., 2017), the third study investigated its impact on the processing of happy and fearful facial expressions (Happy/Fear study; Winker et al., 2018). Here, we investigated the influence of excitatory vs. inhibitory vmPFC-tDCS on facial stimuli with happy and sad facial expressions. As in the Happy/Fear study, we applied a passive viewing paradigm in the MEG scanner combined with two succeeding behavioral tasks (face-morph- and dot-probe task). In sum, our results reveal that, while perception of pleasant scenes and happy faces were designed to evoke approach-like feelings/behaviors and the perception of unpleasant scenes and fearful faces were designed to evoke avoidance-like feelings/behaviors, the feelings/behaviors evoked by perception of sad faces appear to be more complicated than basic approach/avoidance motivation.

In the face-morph task (Figure 2), no significant effects for the shape parameter β were found neither for happy and sad faces nor for a conjoint analysis for pleasant and unpleasant faces. For the perceptual midpoint (mPM), no significant effect was found for happy and sad faces alone. In the conjoint analysis, though, a significant Stimulation × Study interaction was observed. However, while excitatory and inhibitory stimulation had differential effects on happy and fearful faces and thus resulted in a significant main effect of Stimulation in the Happy/Fear study, no main effect of Stimulation was found in the current study. While this indicates a null effect of stimulation, it could also reflect stimulation effects on happy and sad faces in identical directions, i.e., increased approach toward both happy and sad expressions after excitatory compared to inhibitory stimulation. The overall shifted mPM toward a positivity bias in the Happy/Sad compared to the Happy/Fear study (Figure 2B) might support this interpretation. However, a contrast to a so-called sham or placebo stimulation, which was not used here, would be necessary to test this interpretation. Therefore, this interpretation should be investigated further in a future experiment.

For the reaction time data, participants showed significant effects as a function of ambiguity of the presented happy-sad face morphs (Figure 3A). As in the previous Happy/Fear study, we found slower emotional categorization for the most ambiguous stimuli increasing in speed with increasing distinctness. However, prolonged reaction times indicated an overall more difficult emotional categorization of morphed sad and happy compared to morphed fearful and happy faces which again could indicate a relatively reduced distinction of happy and sad compared to happy and fearful faces on the appetitive-avoidance dimension. Interestingly, in the Happy/Sad study, a significant quadratic contrast for Morph Step in the interaction with Stimulation indicated a faster (easier) categorization for highly ambiguous faces after excitatory in comparison to inhibitory stimulation, whereas these differential effects of stimulation vanished with increasing distinctness. While the relatively speeded categorization of happy faces in the Happy/Sad study after excitatory compared to inhibitory stimulation was identical to the effects found for happy faces in the Happy/Fear study, effects of stimulation for sad and fearful faces seemingly differed (Figures 3A,B). Excitatory stimulation led to speeded categorization of sad faces compared to inhibitory stimulation, whereas for fearful faces inhibitory stimulation resulted in relatively speeded reactions compared to excitatory stimulation. This pattern is supported by a significant cubic effect for Morph Step in the interaction Morph Step × Stimulation across both datasets. Thus, it appears that excitatory stimulation induced a positivity bias in the form of faster reactions to happy faces in both studies and inhibitory stimulation induced a negativity bias for fearful faces. However, the effects of stimulation for sad faces might have been more similar to the effects for happy faces and contrary to fearful faces adding up to a null effect across both fearful and sad faces (see Figure 3C). Importantly, as a three-way interaction Morph Step × Stimulation × Study did not reach significance, this inference is rather speculative and can only be indirectly deduced by both the cubic and quadratic effect for the Happy/Fear and the Happy/Sad studies, respectively.

The attentional bias analyses for the dot-probe task (Figure 4) revealed no effects for happy and sad faces alone and thus pointed to the same null findings as in the previous Happy/Fear study. However, when analyzed in combination with the Happy/Fear data, a significant main effect for Stimulation emerged with a relative positive attentional bias after excitatory vmPFC-tDCS and a relative negative attentional bias after inhibitory vmPFC-tDCS. Separated *post hoc*-analyses for pleasant and unpleasant faces, respectively, revealed convergent significant positive attentional biases for happy faces after excitatory compared to after inhibitory stimulation across both studies, Happy/Sad and Happy/Fear, while effects of stimulation differed for sad and fearful faces resulting in no combined effects.

Taken together, results of both behavioral tasks point to a successful manipulation of happy face processing by vmPFC-tDCS in the suggested directions (i.e., excitatory/inhibitory stimulation increases/decreases attentional bias and reaction speed specifically for pleasant stimuli). The modulation of fearful and sad face processing by excitatory and inhibitory vmPFC-tDCS, however, is more complex. This discrepancy might reflect the fact that in contrast to happy and fearful faces, which typically uniquely activate positive or negative feelings in the receiver, respectively, sad faces could potentially activate both, negative empathic distress but also positive feelings of compassion (see “Sad Faces: Approach-Inducing Compassion” for further discussion).

For the event-related MEG data collected during happy and sad face processing, we found a spatio-temporal cluster in left OFC and left temporal pole in a mid-latency time interval (250–290 ms) with a significant interaction effect of Stimulation × Valence (Figure 5B). The pattern of this interaction was consistent with our hypothesis of increased processing of pleasant (here happy faces) relative to unpleasant (here sad faces) stimuli after excitatory compared to after inhibitory stimulation. These left inferior prefrontal areas of activation are involved in the processing of emotionally relevant stimuli: in their review, Rushworth et al. (2011) describe the lateral portions of the OFC (lOFC) to be responsible for linking a reward value to a perceived stimulus. Further support for this comes from Rolls (2007), who describes the OFC to be involved in face-reward links and overall processing of facial expressions. The temporal pole shows, in addition, a coupling between visual stimuli and emotional responses (see Olson et al., 2007, for a review). Both processes are likely to reflect steps in consolidation of stimulus and inherent reward value. In contrast, the vmPFC especially activates as a function of perceived reward itself (Rushworth et al., 2011; Myers-Schulz and Koenigs, 2012). Together with the behavioral findings, these results support our hypothesis of a valence modulation *via* vmPFC-tDCS for happy and sad facial stimuli.

However, three right hemispheric MEG clusters showed effects opposed to our initial hypothesis of relatively increased processing of happy face stimuli compared to sad face stimuli after excitatory compared to inhibitory stimulation. One cluster with a reversed effect pattern for Stimulation × Valence was found in an early time interval of 120–180 ms in right TPJ and right STC (Figure 5A). The analysis across studies revealed two further clusters for interaction effect Stimulation × Valence × Study. During early latency (63–130 ms), a cluster in right occipital cortex showed an activation pattern after excitatory stimulation consistent with our hypothesis for happy vs. fearful faces (Figure 6A). For happy vs. sad faces, a generally higher activation pattern was found for happy faces. This difference, though, was smaller for excitatory compared to inhibitory stimulation. The second cluster in the later interval (350–447 ms; Figure 6B) featured a large cluster across right occipital, parietal, and frontal areas with a simple interaction effect consistent with our hypothesis for happy and fearful faces and a reversed interaction pattern for happy and sad faces. Importantly, in a comparable time interval (357–507) in similar areas, a large interaction effect consistent with our hypothesis was previously found for happy and fearful faces alone (Winker et al., 2018).

The early effects in right occipital cortex (60–130 ms) might reflect early P1 component differences. The P1 has been shown to reflect emotional relevance of presented stimuli, e.g., faces (Holmes et al., 2008; Bublatzky and Schupp, 2012). After excitatory stimulation compared to after inhibitory stimulation, the difference between both emotion conditions decreased, which could imply first signs for a relative increase in attention allocation to sad faces. As a possible continuation of this early effect, the cluster during 120–180 ms yielded an even more pronounced pattern. The cluster in STC and TPJ showed higher activations for sad faces vs. happy faces after excitatory stimulation and vice versa after inhibitory stimulation. Involvement of STC could point to an activation of STS, which processes specifically changeable aspects of faces, e.g., emotional facial expressions (Haxby et al., 2000). This effect reappeared again during late-latency and mirrored possible LPP modulation with still increased activation for sad faces relative to happy faces after excitatory compared to inhibitory stimulation. The hypothesized spatio-temporal cluster in occipito-temporal and parietal areas with an increased activation for happy faces compared to sad faces after excitatory and vice versa after inhibitory stimulation, however, was not observed. Location and timing for the hypothesized and the actual finding point to a more ventral and a more dorsal, respectively, activation pattern for a modulated LPP (Sabatinelli et al., 2013).

Together, all three right hemispheric spatio-temporal clusters revealed a relatively stronger neural activity for sad face processing after excitatory compared to after inhibitory vmPFC stimulation. This is incompatible with our initial hypothesis of a reduced negative emotional value of sad faces (i.e., reduced initiation of avoidance) after excitatory compared to inhibitory stimulation and might, in contrast, indicate an increased approach toward sad faces after excitatory stimulation.

### Sad Faces: Approach-Inducing Compassion

In this study, we chose to extend our findings on the modulation capability of vmPFC to the investigation of sad face processing. This choice was motivated by the fact that sad facial expressions are often employed in the investigation of mood disorders, e.g., MDD. However, results of studies investigating sad face processing in MDD patients point to some interpretive difficulties. On the one hand, some research groups reported increased attention allocation in MDD patients specifically to sad faces compared to healthy controls (Gotlib et al., 2004; Joormann and Gotlib, 2007). Surguladze et al. (2004), on the other hand, refer to a decreased performance in sad face categorization specifically for MDD patients which occurred, though to a lesser degree, for happy faces, too. These latter results point to a relative blunting of affect recognition rather than a negativity bias in MDD. Another study (Gur et al., 1992) reports mixed results with MDD patients, yielding a hypothesized negativity bias with happy faces being categorized more often as neutral and neutral faces more often as sad faces. Nevertheless, with increasing symptom severity, MDD patients showed higher rates of sad faces being falsely categorized as neutral faces. Therefore, the assumption of a negativity bias of sad face processing in MDD patients might be an oversimplification that has to be regarded tentatively. Moreover, literature on event-related electrophysiological data for happy and sad face processing is rather scarce and also reveal mixed results. To our knowledge, only one study has reported event-related potential (ERP) differences between happy and sad face processing in healthy participants (Calvo and Beltrán, 2013) while other studies have not found any ERP distinctions (Herrmann et al., 2002; Balconi and Pozzoli, 2003; Eimer et al., 2003; Smith et al., 2013).

An explanation for these inconsistent findings in sad face processing could lie in the inherent ambiguity of sadness in social communication. The communication of happiness by happy facial expressions is typically a pleasant state for both the sender and the receiver. In the same vein, communication of fear by fearful expressions is typically an aversive state for both sender and receiver as it should communicate potential environmental danger^4^. The communication of suffering, such as pain or sadness, however, can at times be more nuanced (Singer and Klimecki, 2014). As experienced by oneself, pain and sadness can be straightforwardly described as unpleasant emotions. In the receiver, a facial expression communicating pain or sadness can induce unpleasant feelings such as empathic distress evoking avoidance and withdrawal. However, pain or sadness expressing faces can also induce empathic feelings of caring or compassion evoking comforting and thus approach-like behavior with the motivation to improve the other’s wellbeing.

The right hemispheric pattern found in the MEG analysis might support the *post hoc*-hypothesis that excitatory compared to inhibitory stimulation increased the empathy-related compassion inducing value of sad faces (Singer and Klimecki, 2014). This interpretation is supported by two studies showing that short-term compassion training of several days was, in fact, able to increase positive affect and increase vmPFC and striatum activations toward stimuli depicting suffering from others (Klimecki et al., 2013, 2014). Moreover, relatively increased activation of sad face processing after excitatory compared to inhibitory vmPFC-tDCS was also found in areas covering right STC and right TPJ. Especially these areas were often found to be involved in Theory of Mind (ToM)-processes (for reviews, see Shamay-Tsoory, 2011; Bernhardt and Singer, 2012). Interestingly, ToM plays a vital role in empathy as it allows a person to cognitively adopt the perspective of others. The vmPFC is strongly interconnected with the TPJ and importantly—with regard to ToM—involved in self-other differentiation, e.g., emotion perception in others (Shamay-Tsoory, 2011). Thus, excitatory compared to inhibitory vmPFC stimulation might have increased compassion related approach toward sad faces as revealed in all right hemispheric clusters. A broader interpretation would be a more thorough evaluation of ambiguous sad faces in order to decide whether to react with approach-like compassion or withdrawal-like empathic distress. With regard to our behavioral data, we were able to find an induced positivity bias for happy faces after excitatory compared to inhibitory stimulation for the dot-probe task and reaction time data of the face-morph task. However, the face-morph task effects for happy and sad faces were quite similar and thus might better support the processing of sad faces in favor of an approach-like perception.

### Limitations

To reduce intra-individual variance of psychological and physiological factors—like vigilance, mood, hormonal status et cetera—across days and allow a direct comparison with results of our preceding studies, we again applied a within-subjects design with a delay of around 90 min between both stimulation types. Although aftereffects of 9 min anodal and cathodal tDCS of the motor cortex typically vanish within 90 min (Nitsche et al., 2005; Monte-Silva et al., 2010), it is unknown if aftereffects for vmPFC regions might be prolonged. With the assumption of simply additive effects and based on the applied balancing of stimulation order, any residual superposition of opposite effects should have just reduced effect sizes to a rather small degree. Monte-Silva et al. (2010), however, showed that a repetition of 9 min cathodal stimulation after 3 h delay could attenuate inhibitory aftereffects indicating non-linear effects. Replication studies with significant longer delays between both stimulation types should test if stimulation delay has a relevant impact on the stimulation × valence interactions reported in this and the preceding studies.

We did not measure individual empathy of study participants toward feelings of suffering in other individuals. Covariations of effect size with individual empathy may have supported our *post hoc*-interpretation that approach related feelings of compassion might be modulated by vmPFC-tDCS. Future studies targeting this hypothesis might choose a range of low to high compassion participants or even modulate feelings of compassion within subjects by, for instance, choosing sad faces from loved ones as compared to strangers.

As in the previous studies, we directly compared the effects of excitatory and inhibitory stimulation instead of comparing active and “sham” stimulation. A sham stimulation could have supported our hypothesis that both happy and sad face processing is shifted toward a positivity bias by excitatory stimulation. Future studies probably using a local anesthetic at the stimulation sites (McFadden et al., 2011), could prevent a conscious distinction between real and sham stimulation^5^ and might provide further evidence supporting this interpretation. We selected the facial stimuli used in the Happy/Fear and Happy/Sad studies based on optimal performance of the morphing procedure and best authenticity of the perceived emotional expression (happiness, fear, sadness). However, other factors like attractiveness or arousal might have differed across categories and might thus have influenced the results. Hence, future studies should preclude these uncertainties by additional control of the experimental stimuli.

After stimulation, all participants were first measured in the MEG and completed the two behavioral tasks thereafter. Thus, with decreasing aftereffects, the effects of stimulation on behavior should have been relatively reduced compared to the effects on the neural measures. Future studies should either consider a complete balancing of order or conduct additional studies assessing post-stimulation effects on behavioral data only.

## Conclusion

The present findings may be helpful in suggesting goals for future research on vmPFC related tasks, for instance, emotion-, reward-, empathy- or pain-processing. As in three previous experiments (Junghofer et al., 2017; Winker et al., 2018), vmPFC-tDCS here successfully induced a positivity bias specifically for pleasant emotional stimuli, further supporting the role of the vmPFC as a valence driven region which can be successfully modulated by excitatory and inhibitory tDCS. However, the somewhat contradictory electrophysiological findings highlight the need for more investigations on the perception of sad facial expressions. The underlying ambiguity of sadness, which can induce both approach- and withdrawal-like behavior simultaneously when observed in others, renders an investigation of this emotional expression difficult. Specifically, regarding this ambiguity, the interpretation of disrupted sad face processing in mood-disordered individuals and directed hypotheses for therapy induced modulations of sad face processing is rather complex. Thus, more research on the respective impact of the vmPFC on underlying components of sad-face processing appears necessary. Furthermore, a general modulation of valence processing in clinical samples such as MDD patients would be useful. In fact, a first clinical pilot trial with repeated application of excitatory vmPFC-tDCS over the course of 2 weeks yielded promising preliminary results (Rehbein et al., 2017). Nevertheless, with the vmPFC as a hub of many processes initiating different behaviors, additional control studies should be considered as well. An increase in stimulation focality and a disentanglement of the variety of processes affected by vmPFC-tDCS could help to improve selectivity and foster the development of a successful adjunct to clinical therapy in the future.

## Ethics Statement

Ethics Committee of the Department of Psychology and Sports Science at the University of Muenster, Germany.

## Author Contributions

MJ, CW, MR, JM, CHW, VA and KR conceptualized the work. CW, MR, MJ, and JM implemented the study design. MD, JM, and CW conducted data acquisition. CW, MR, MJ, MD and JM analyzed and interpreted the data. CW, MJ, DS and MR drafted the work. MD, JM, KR, CHW, and VA revised the work.

## Conflict of Interest Statement

The authors declare that the research was conducted in the absence of any commercial or financial relationships that could be construed as a potential conflict of interest.

## Footnotes

^1^who.int/news-room/fact-sheets/detail/depression

^2^users.aber.ac.uk/bpt/jpsychomorph

^3^emegs.org

^4^This differs if fear in the sender is induced by the receiver him- or herself, e.g., by an angry expression of the receiver toward the sender. However, as the receiver in our studies, i.e., the study participants, can be expected to only passively and not actively communicate with the senders (the stimuli), we could assume that fearful expressions should evoke only negative and not positive feelings in the receiving subject.

^5^Sensations below anode and cathode during active stimulation cannot be differentiated reliably, while both can easily be distinguished from a sham stimulation.
