# Supplementary material for: Noninvasive Stimulation of the Ventromedial Prefrontal Cortex Indicates Valence Ambiguity in Sad Compared to Happy and Fearful Face Processing
Source: Front Behav Neurosci. 2019 May 16;13:83. doi: 10.3389/fnbeh.2019.00083 (PMC6532016; doi:10.3389/fnbeh.2019.00083)
Supplement: Supplementary file 1 [file Table_1.DOCX]

Supplementary Material

#### Goodness of fit (GoF): happy and sad faces

As the GoF parameter violated the assumption of normality, we again conducted a Wilcoxon signed-rank test. Comparison of conditions Excitatory and Inhibitory resulted in a significant effect (*Z* = -2.83, *p* = .005) with the data assessed after inhibitory stimulation showing a higher GoF value for the Weibull function and thus better fit than for the post-excitatory data.

#### Goodness of fit (GoF): pleasant and unpleasant faces

For the GoF parameter comparison between pleasant and unpleasant face stimuli, we again ran a Wilcoxon signed-rank test resulting in a significant effect (*Z* = -3.16, *p* = .002) with the same direction as reported above (i.e. better Weibull fit after inhibitory compared to excitatory stimula­tion). Here, as in the Happy/Sad study, a post hoc-analysis of the Happy/Fear data pointed toward, by trend (*Z* = -1.81, *p* = .071), higher GoF values after inhibitory tDCS. Results of the GoF parameter for study Happy/Fear differ in comparison to those previously reported (Winker et al., 2018) as we here applied a different preprocessing routine. In this study, participants were excluded from all analyses that comprised values of the Weibull Fit if they exceeded a GoF value > 3 x SD. This step was omitted previously.

**Discussion**

Investigation of the GoF parameter for happy-sad face morphs and happy-fear face morphs revealed better Weibull function fits after inhibitory compared to after excitatory stimulation. While this finding corroborates our interpretation that vmPFC-tDCS modulates behavior, the underlying mechanisms leading to this unexpected finding remain unclear and await further studies with the inclusion of a sham stimulation condition.
